# Supplementary material for: Cryo-EM structure of severe fever with thrombocytopenia syndrome virus
Source: Nat Commun. 2023 Oct 10;14:6333. doi: 10.1038/s41467-023-41804-7 (PMC10564799; doi:10.1038/s41467-023-41804-7)
Supplement: Supplementary file 1 — Supplementary Information [file 41467_2023_41804_MOESM1_ESM.pdf]

Supplementary Information

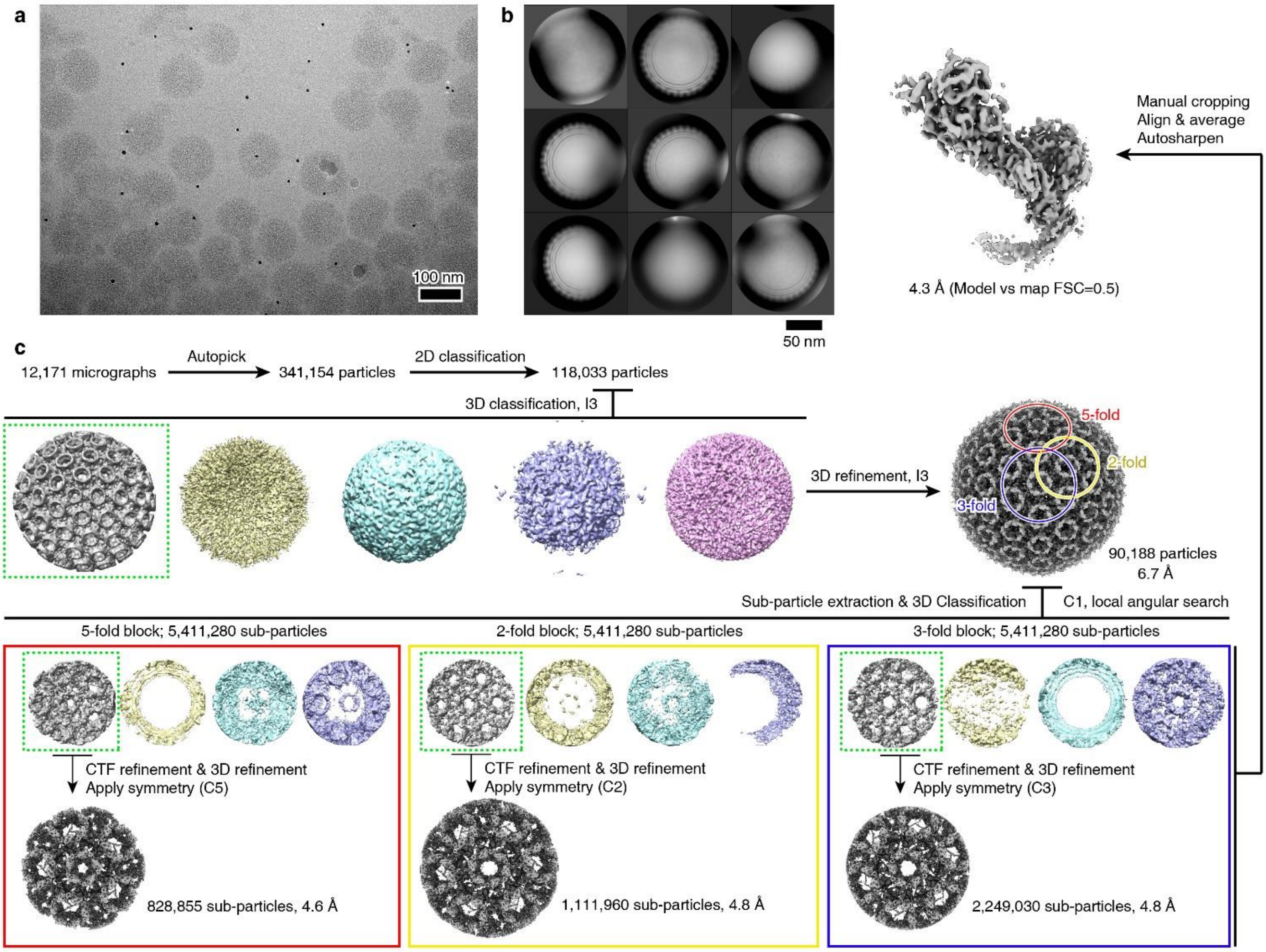

Supplementary Fig. 1. Cryo-EM image processing workflow for SFTSV viral particles. a, A representative cryo-EM micrograph of SFTSV virions. A total of 12,171 micrographs were collected for this study. **b,** Gallery of 2D class average images of SFTSV, showing the spherical morphology of viral particles with smeared density in local regions. **c,** The workflow of image processing and 3D reconstruction.

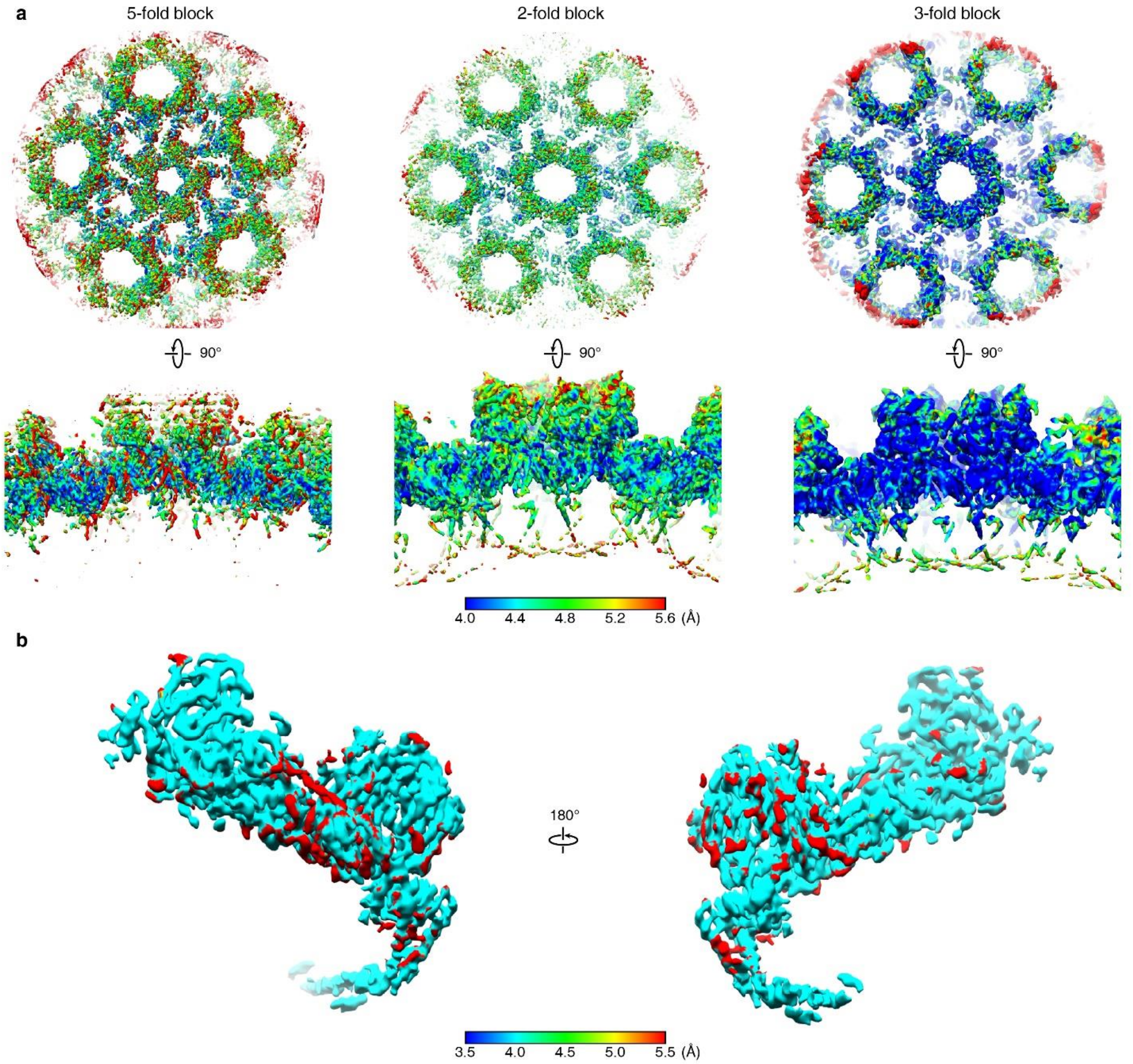

Supplementary Fig. 2. Local resolution assessment of the density maps. a, The local resolution maps of the three block reconstructions. The best resolved regions reached 4 Å local resolution. **b,** After further averaging of individual Gn/Gc heterodimers, most regions of the ectodomain showed close to 4 Å local resolution.

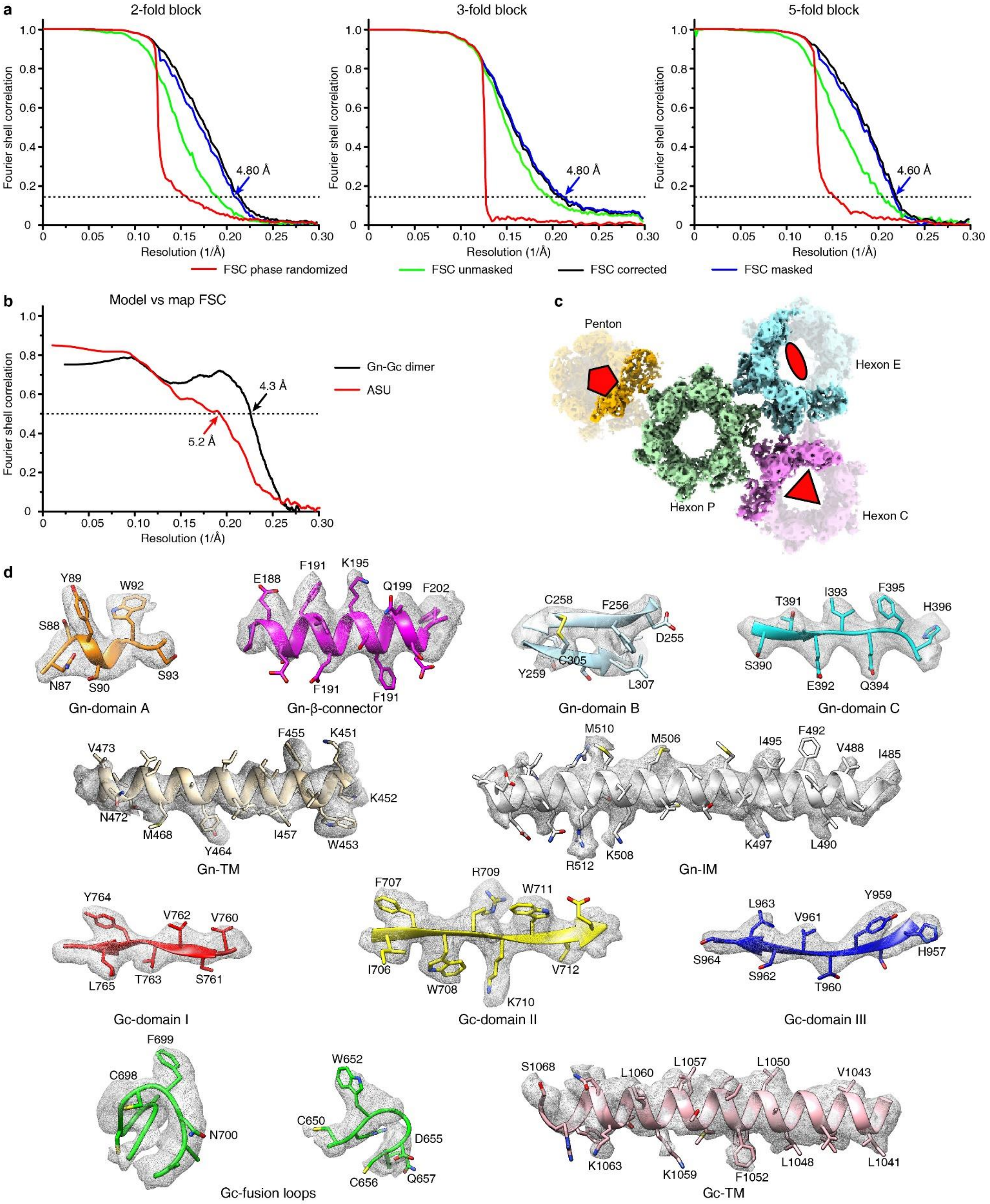

Supplementary Fig. 3. FSC curves and representative density map in selected regions. a, FSC curves for each block of locally reconstructed density map. The gold-standard FSC=0.143 cut-off value is indicated by dashed lines. **b,** The model vs map FSC curves for the final atomic models and density maps. **c,** The composite density map of an ASU, assembled with the locally reconstructed block maps. The 5-, 2- and 3-fold symmetry axes are represented by a pentagon, oval and triangle, respectively. **d,** Representative density map and atomic models in selected regions. The bulky side chains could be resolved in some regions.

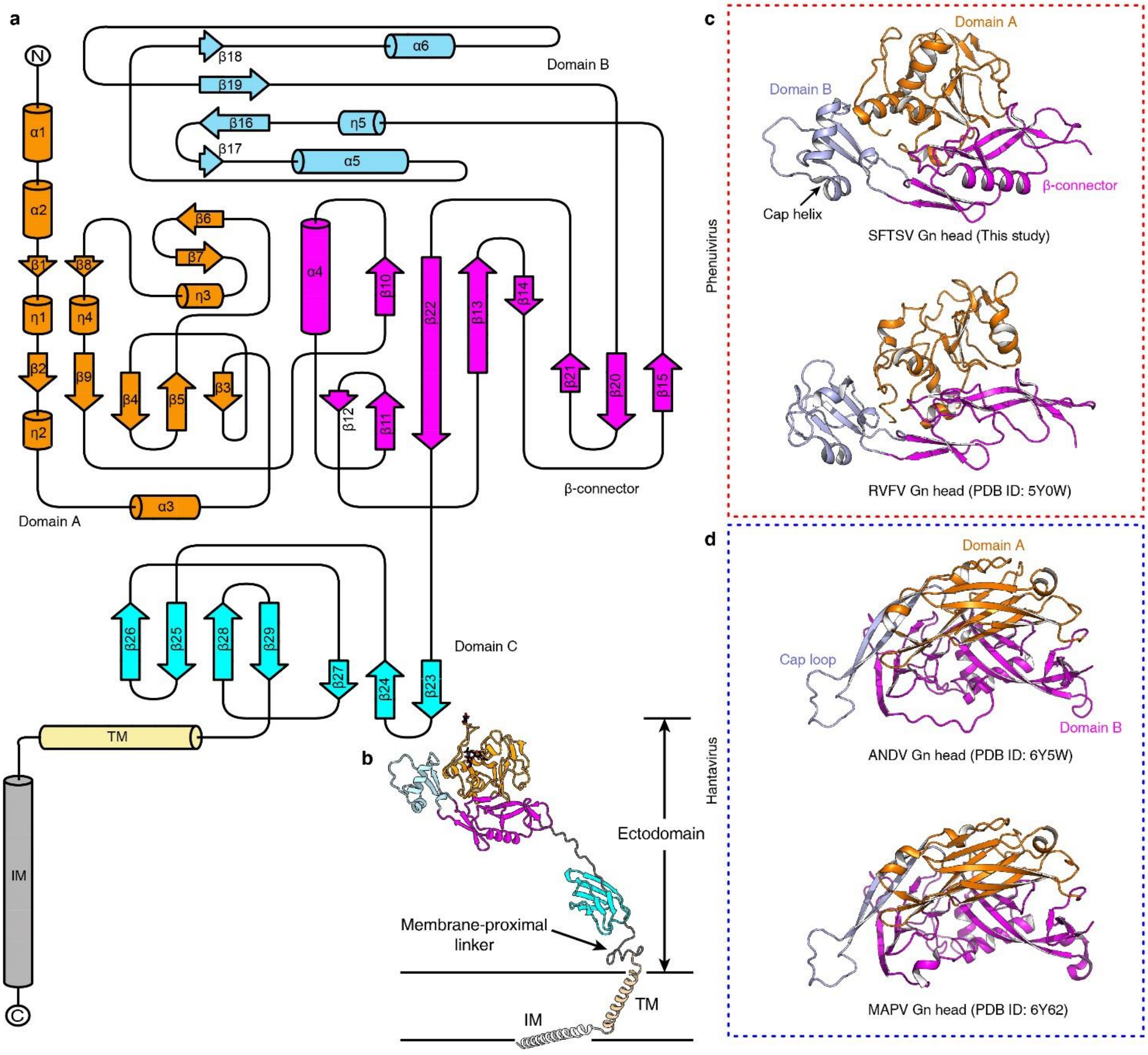

Supplementary Fig. 4. Structural comparison of different bunyavirus Gn proteins. a, Topology diagram of SFTSV Gn protein, colored by domains. **b,** Overall structure of SFTSV Gn, colored by domains. **c-d,** Structures of the Gn head from different bunyaviruses, with the same color code as in **(a)**. The 3-domain architecture is conserved among different viruses.

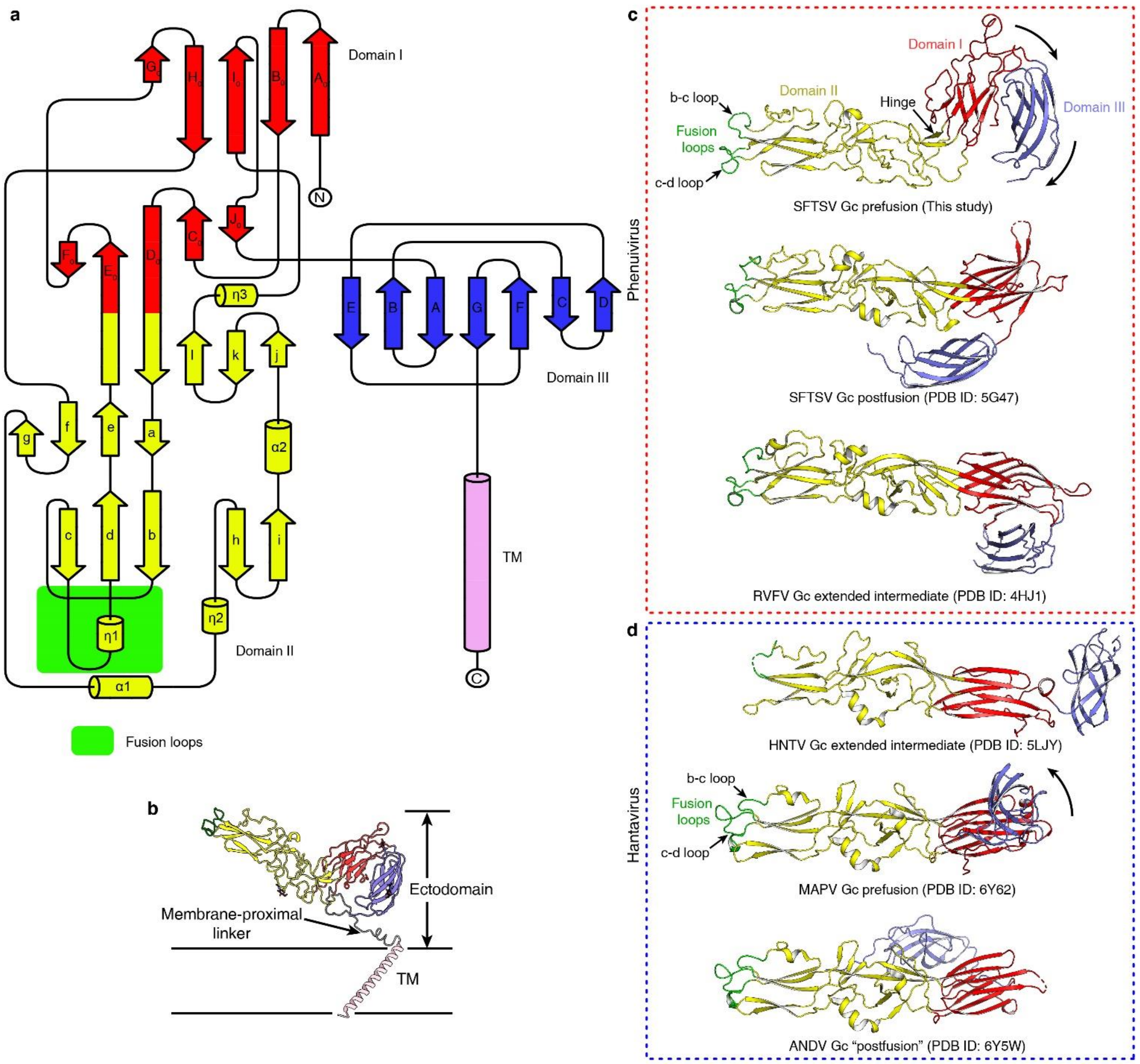

Supplementary Fig. 5. Structural comparison of different bunyavirus Gc proteins. a, Topology diagram of SFTSV Gc protein, colored by domains. **b,** Overall structure of SFTSV Gc, colored by domains. **c-d,** Multiple conformations of the Gc protein from different bunyaviruses, with the same color code as in **(a)**. The fusion loops are highlighted in green. All these proteins display typical features of class II viral fusion proteins, and adopt different conformations corresponding to different states in membrane fusion. The directions of conformational changes from prefusion to postfusion states are indicated by arrows.

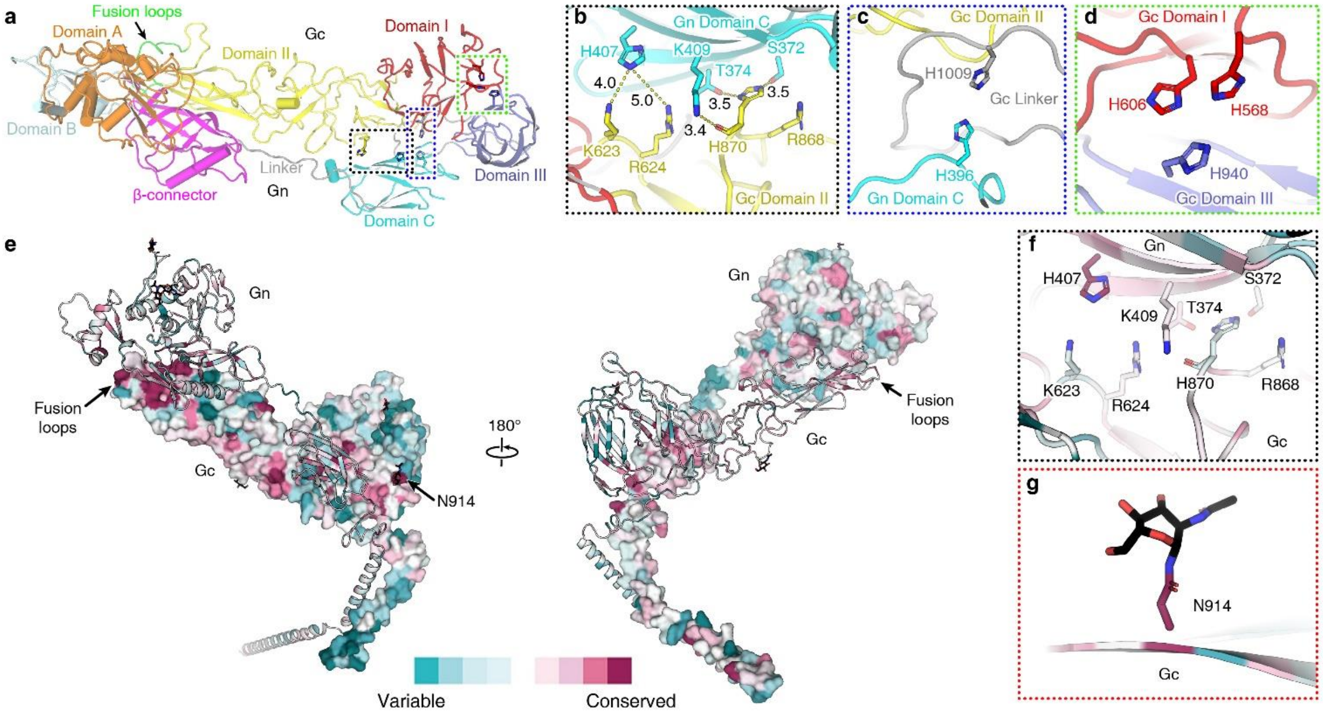

Supplementary Fig. 6. pH-sensitive interfaces within SFTSV Gn/Gc heterodimer and the conformational changes for membrane fusion. a, Structure of Gn/Gc heterodimer colored by domains. The potential pH-sensitive interfaces are indicated by dashed boxes. **b-d,** Close-up views of the pH-sensitive interfaces as indicated in **(a)**. Multiple histidines are observed in each interface that may serve as pH-sensors to induce conformational changes in low-pH conditions. In **(b)**, the potential hydrogen bond interactions are shown as dashed lines, labeled with the distance (in Å). **e,** Conservation analysis of Gn/Gc heterodimer among different phleboviruses and SFTSV. Only some small local regions display high degree of conservation. **f-g,** Residue H407 on one of the pH-sensitive interfaces and the glycans on residue N914 are highly conserved for all phleboviruses (and SFTSV). These two sites are less conserved for the *Bandavirus* genus, which are not included in this analysis.

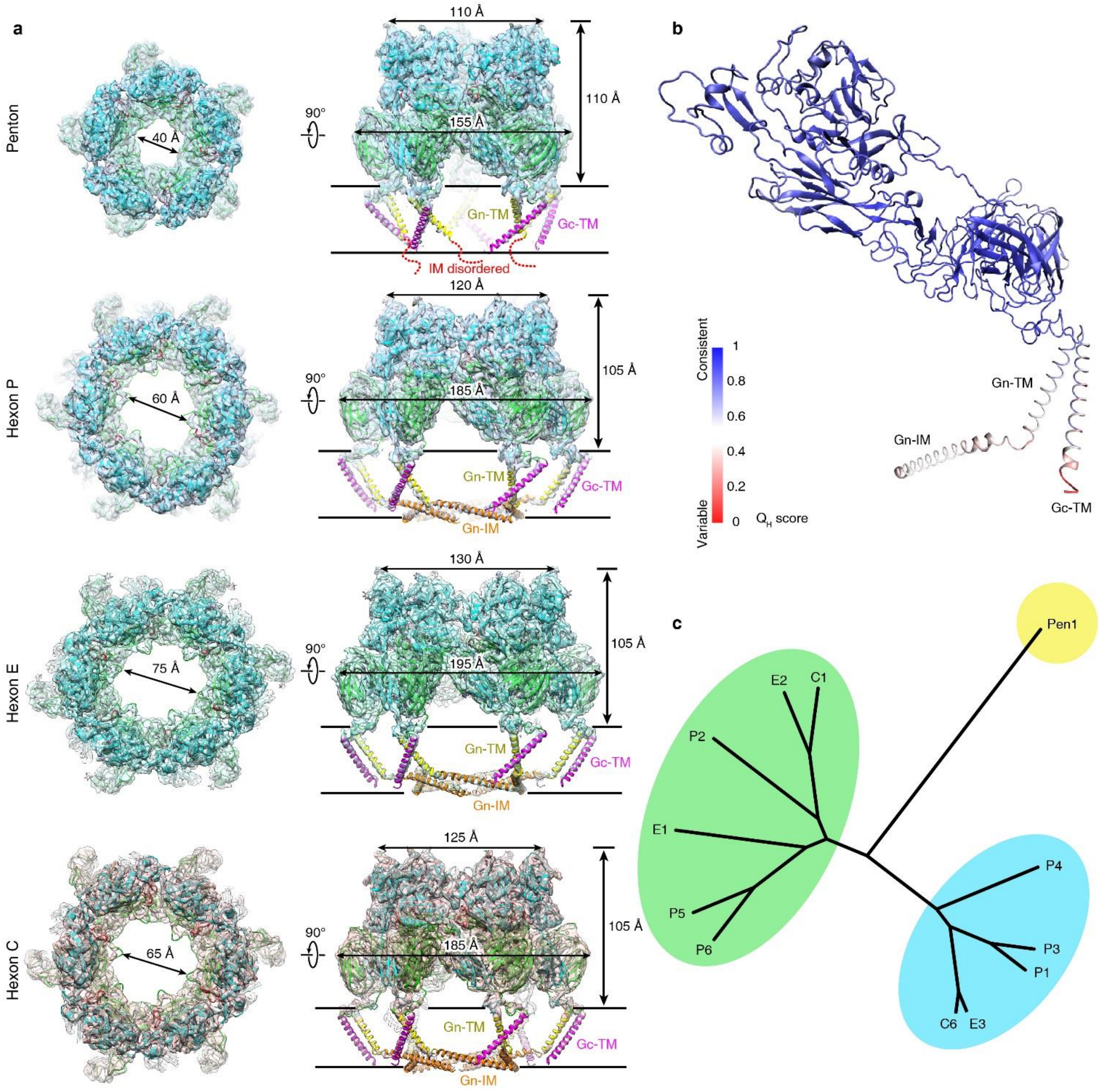

Supplementary Fig. 7. Flexibility of different Gn/Gc conformers in SFTSV. a, Structural comparison of penton and three types of hexons of SFTSV. The overall architecture is highly similar, with some variations in local conformations. **b,** Superposition of the 12 Gn/Gc heterodimers within an ASU, colored by the degree of conformational variations. The conformations of the ectodomains are very similar, while the orientations of the TM regions display obvious variations. A Q_H score of 0 indicates the structures are absolutely not similar, and a score of 1 indicates the structures are identical. **c,** Conformation clustering of the 12 Gn/Gc heterodimers within an ASU. The 11 conformers from hexons cluster into two groups, with the largest difference from the penton conformer. The individual Gn/Gc heterodimers within an ASU are numbered with the same scheme as Fig. 2d.

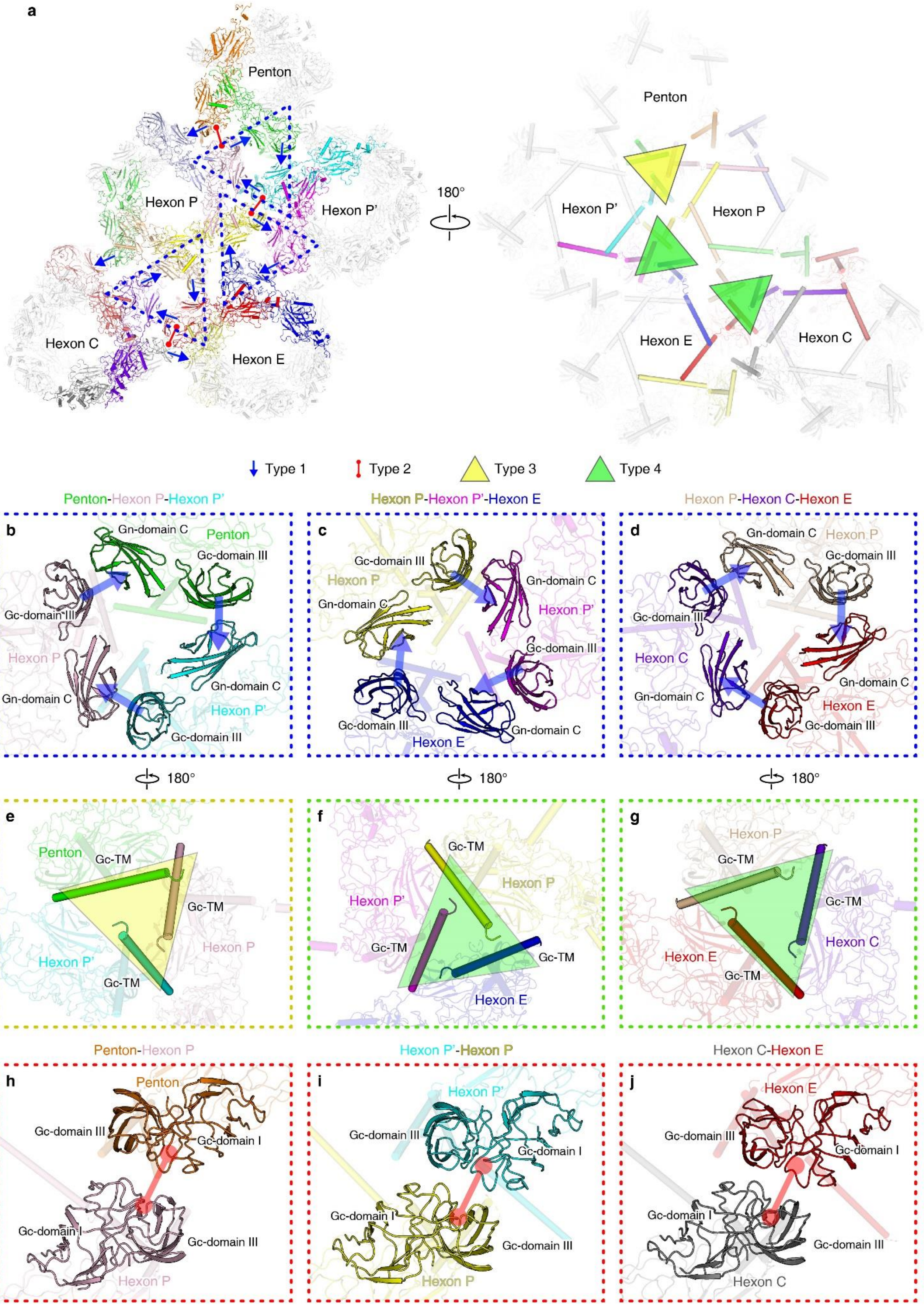

Supplementary Fig. 8. Molecular contexts of inter-peplomer interactions. a, Schematic representation of the inter-peplomer interactions within an ASU. Each Gn/Gc heterodimer at the interface is represented with a unique color. The four types of interactions are labeled on the structure as the legend below. The dashed triangles indicate the closed networks created by three sets of type 1 interaction among three adjacent peplomers. **b-d,** Zoom-in views of type 1 interactions in the context of three adjacent

peplomers. The blue arrows are set transparent to reveal the polarity of the interface, with the Gc domain III of one peplomer interacting with the Gn domain C from the neighboring peplomer. **e-g,** Close-up views of type 3 and type 4 interactions, which are located under the network of type 1 interactions. **h-j,** Close-up views of the contexts of type 2 interactions. This interface consists of the domain I and domain III of Gc from two neighboring peplomers, arranged in a head-to-head orientation, which are represent ed with red dumbbells to reveal the symmetry of interactions.

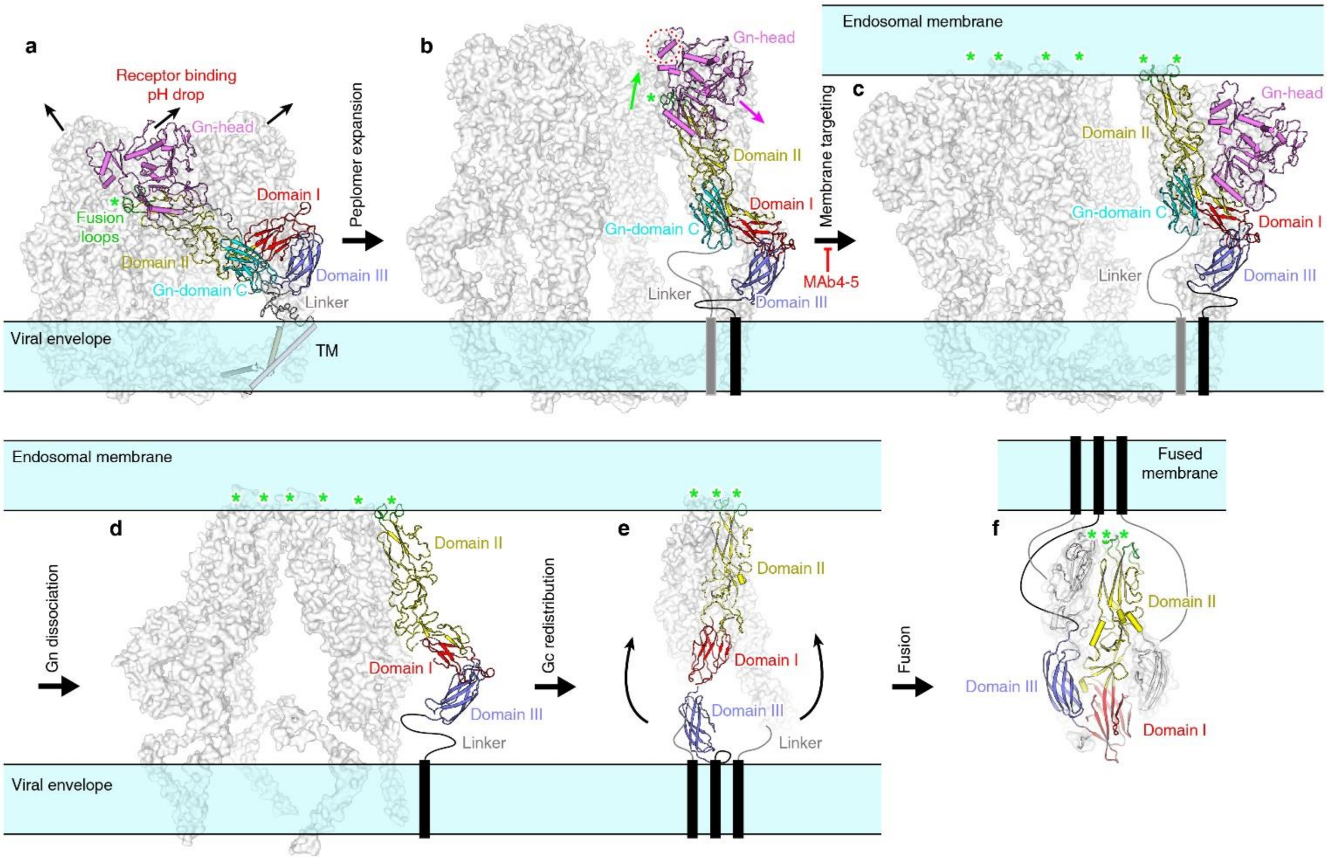

Supplementary Fig. 9. A proposed model of peplomer conformational changes that mediate membrane fusion, depicted in the context of a hexon. For clarity, one of the Gn/Gc heterodimers is shown in cartoons and colored by domains, with the others shown as white surface. The fusion loops are highlighted by green asterisks and the conformational changes are indicated by arrows. Initially, the viral particles are internalized into the endosome after binding to receptors[14]. **a,** With the acidification of the endosome, the peplomers begin to expand triggered by the protonation of histidines at the pH-sensitive interfaces. **b,** The ectodomains of Gn/Gc heterodimer will tilt to higher angles relative to the viral envelope, getting closer to the endosomal membrane. **c,** The Gn head then retracts to a lower position and allows the fusion loops of Gc to reach out further and insert into the endosomal membrane[43]. **d-e,** Subsequently, the entire Gn subunit dissociates from Gc and allows the Gc subunits to reassemble into homotrimers in the extended conformation. **f,** With further conformational changes of Gc, domain III folds back towards domain II to form a hairpin configuration, which drags the viral and endosomal membranes to close proximity and induce fusion. In **(b)**, One of the epitopes in Gn domain B (targeted by MAb4-5) is only exposed when the peplomer expands, highlighted with a dashed circle. MAb4-5 (ref.[25]) probably targets this intermediate state to lock the conformation of Gn/Gc heterodimer to prevent the exposure of fusion loops, thus inhibiting membrane fusion.

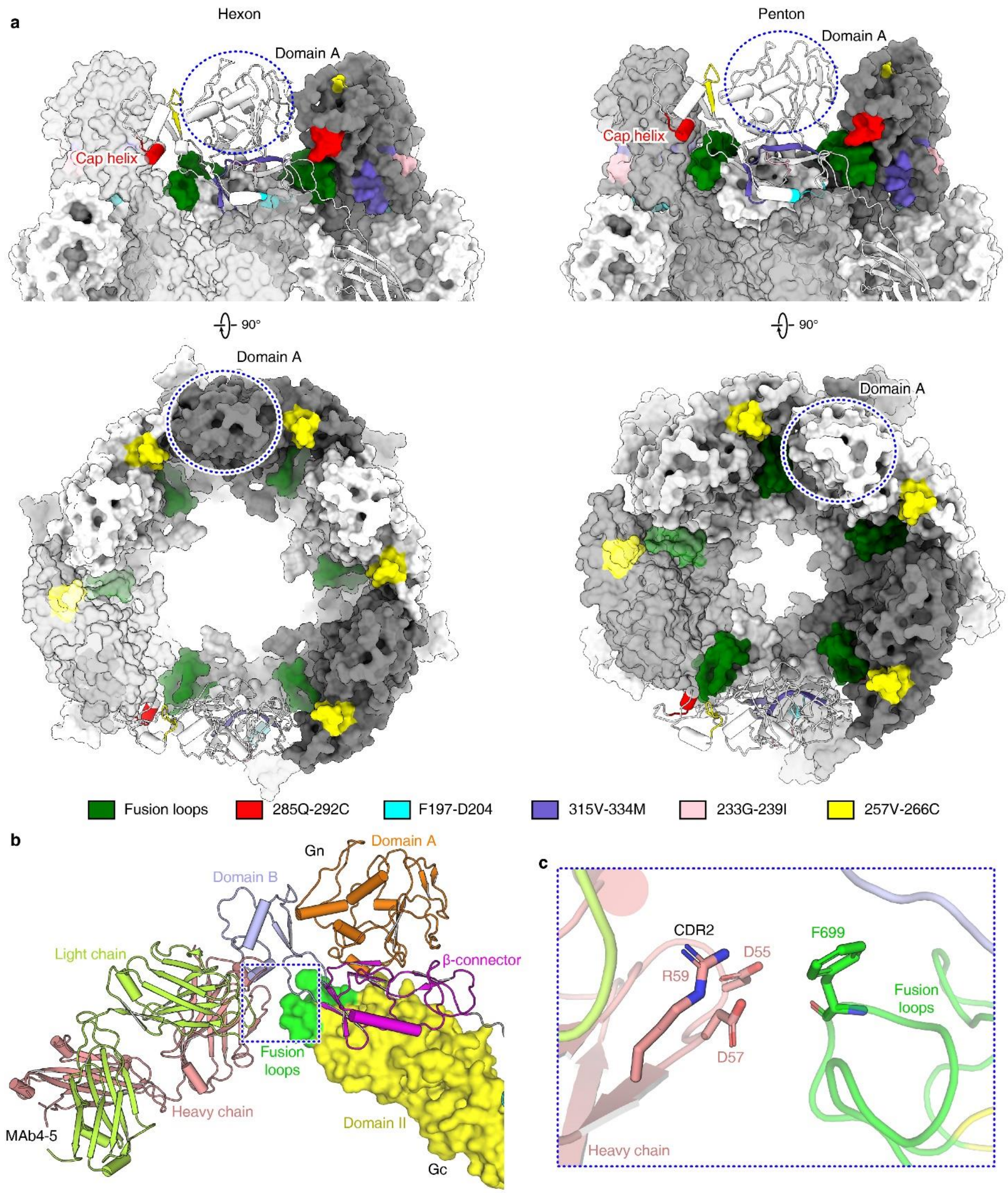

Supplementary Fig. 10. Neutralizing epitopes in SFTSV envelope glycoproteins. a, Spatial locations of the known neutralizing epitopes in the context of penton and hexon peplomers. The epitopes identified by previous studies[24-26] are mapped onto the structures of SFTSV glycoproteins, each represented with a different color as shown in the legend below. For clarity of visualization, one Gn subunit is shown in cartoons, with the other subunits shown as surface. Domain A of one Gn subunit is highlighted with a dashed oval, of which the top surface is fully exposed and potentially involved in binding host receptors. **b,** Docking of MAb4-5 onto the structure of SFTSV Gn/Gc heterodimer in the prefusion conformation. The crystal structure of MAb4-5 bound to Gn head (PDB ID: 5Y11) was aligned to Gn/Gc heterodimer by superimposing the Gn head. The heavy chain of MAb4-5 mainly binds to Gn domain B, and may also interact with the fusion loops of Gc, highlighted in a dashed box. **c,** Close-up view of the interface between MAb4-5 and the Gc fusion loops. The potential interacting residues are shown as sticks. CDR2, complementarity-determining region 2.

Supplementary Table 1. Cryo-ET data collection and processing statistics.

	SFTSV virion
**Tilt series data collection**	
Microscope	Titan Krios
Magnification	53,000
Detector	Gatan K3
Voltage (kV)	300
Defocus range (µm)	-1.5 to -4.0
Pixel size (Å)	1.68
Collection scheme	Dose-symmetric
Tilt range (°)	-60 to 60
Tilt step (°)	4
Electron dose per tilt ($e^-/Å^2$)	31 (0° tilt); 3.46 (other tilt angles)
Frames per tilt	90 (0° tilt); 10 (other tilt angles)
Cumulative electron dose ($e^-/Å^2$)	135
**Subtomogram averaging**	
Particle picking method	Template matching
Template structure (EMDB)	EMD-4197
No. of tomograms	118
No. of final subtomograms	15,535
Symmetry imposed	C6
Map resolution (Å)	10.9
FSC threshold	0.143
Modelling strategy	Rigid body fit

Supplementary Table 2. Cryo-EM data collection, reconstruction, and refinement statistics.

**Data collection and processing**	SFTSV virion	2-fold block	3-fold block	5-fold block
Microscope	Titan Krios	-	-	-
Magnification	53,000	-	-	-
Detector	Gatan K3	-	-	-
Voltage (kV)	300	-	-	-
Electron exposure ($e^{-}/Å^2$)	60	-	-	-
Defocus range (μm)	-1.0 to -3.5	-	-	-
Pixel size (Å)	1.68	-	-	-
Symmetry imposed	I3	C2	C3	C5
Final particle images (no.)	90,188	1,111,960	2,249,030	828,855
Map resolution (Å)	6.7	4.80	4.80	4.60
FSC threshold	0.143	0.143	0.143	0.143
Map resolution range (Å)	6.0-10.0	4.0-6.0	4.0-6.0	4.0-6.0
Map sharpening *B* factor ($Å^2$)	-468	-298	-197	-300

**Refinement**	Gn/Gc heterodimer	ASU composite map
Initial model used (PDB code)	5Y10, 5G47	Gn/Gc heterodimer
Model resolution (Å)	4.3	5.2
FSC threshold (model vs map)	0.5	0.5
Map correlation coefficient		
Whole unit cell	0.67	0.710
Around atoms	0.73	0.713
Model composition		
Non-hydrogen atoms	7,729	91,976
Protein residues	1,010	12,029
Glycan residues	6	66
B factors ($Å^2$)		
Protein	37	230
Glycans	57	223
R.m.s. deviations		
Bond lengths (Å)	0.003	0.002
Bond angles (°)	0.66	0.47
**Validation**		
MolProbity score	2.11	2.32
Clashscore	9.06	6.90
Poor rotamers (%)	0.23	4.18
Ramachandran plot		
Favored (%)	86.68	92.35
Allowed (%)	13.12	7.52
Disallowed (%)	0.20	0.13
Ramachandran Z score		
Whole	-2.78 (N=1006)	-3.37 (N=11959)
Helix	0.21 (N=147)	1.12 (N=1919)
Sheet	-1.27 (N=228)	-1.87 (N=2611)
Loop	-2.74 (N=631)	-3.55 (N=7429)
