# Supplementary material for: Cryo-EM structure of severe fever with thrombocytopenia syndrome virus
Source: Nat Commun. 2023 Oct 10;14:6333. doi: 10.1038/s41467-023-41804-7 (PMC10564799; doi:10.1038/s41467-023-41804-7)
Supplement: Supplementary file 3 — Reporting Summary [file 41467_2023_41804_MOESM3_ESM.pdf]

nature portfolio

Corresponding author(s): Wei Liu, Jianxun Qi, Quan Liu, Ming Liao, Chang Li

Last updated by author(s): Sep 4, 2023

Reporting Summary

Nature Portfolio wishes to improve the reproducibility of the work that we publish. This form provides structure for consistency and transparency in reporting. For further information on Nature Portfolio policies, see our Editorial Policies and the Editorial Policy Checklist.

Statistics

For all statistical analyses, confirm that the following items are present in the figure legend, table legend, main text, or Methods section.

n/a	Confirmed	
☐	☒	The exact sample size (*n*) for each experimental group/condition, given as a discrete number and unit of measurement
☐	☒	A statement on whether measurements were taken from distinct samples or whether the same sample was measured repeatedly
☐	☒	The statistical test(s) used AND whether they are one- or two-sided<br>*Only common tests should be described solely by name; describe more complex techniques in the Methods section.*
☒	☐	A description of all covariates tested
☒	☐	A description of any assumptions or corrections, such as tests of normality and adjustment for multiple comparisons
☐	☒	A full description of the statistical parameters including central tendency (e.g. means) or other basic estimates (e.g. regression coefficient) AND variation (e.g. standard deviation) or associated estimates of uncertainty (e.g. confidence intervals)
☐	☒	For null hypothesis testing, the test statistic (e.g. *F*, *t*, *r*) with confidence intervals, effect sizes, degrees of freedom and *P* value noted<br>*Give P values as exact values whenever suitable.*
☒	☐	For Bayesian analysis, information on the choice of priors and Markov chain Monte Carlo settings
☒	☐	For hierarchical and complex designs, identification of the appropriate level for tests and full reporting of outcomes
☒	☐	Estimates of effect sizes (e.g. Cohen's *d*, Pearson's *r*), indicating how they were calculated

Our web collection on statistics for biologists contains articles on many of the points above.

Software and code

Policy information about availability of computer code

Data collection	SerialEM-3.8 was used for cryo-EM data collection
Data analysis	MotionCor2; GCTF-0.50; IMOD-4.9; emClarity-1.3.0; emClarity-1.4.3; Gautomatch-0.53; Relion-3.0; Relion-4.0; ResMap(1.1.4); Chimera(1.11); ChimeraX(1.5); COOT(0.8.1); Phenix(1.20); MolProbilty(4.3.1); PyMOL(2.0); VMD(1.9.3); FigTree(1.4.4); MultiSeq-2.0

For manuscripts utilizing custom algorithms or software that are central to the research but not yet described in published literature, software must be made available to editors and reviewers. We strongly encourage code deposition in a community repository (e.g. GitHub). See the Nature Portfolio guidelines for submitting code & software for further information.

Data

Policy information about availability of data

All manuscripts must include a data availability statement. This statement should provide the following information, where applicable:

- Accession codes, unique identifiers, or web links for publicly available datasets
- A description of any restrictions on data availability
- For clinical datasets or third party data, please ensure that the statement adheres to our policy

The cryo-EM density maps and associated model coordinates generated in this study have been deposited to the Electron Microscopy Data Bank (EMDB) and Protein Data Bank (PDB) databases, respectively. The accession codes are listed as follows: EMD-35152 [https://www.ebi.ac.uk/emdb/EMD-35152] (Full virion icosahedral reconstruction), EMD-35176 [https://www.ebi.ac.uk/emdb/EMD-35176] (2-fold block local reconstruction), EMD-35177 [https://www.ebi.ac.uk/emdb/

EMD-35177] (3-fold block local reconstruction), EMD-35178 [https://www.ebi.ac.uk/emdb/EMD-35178] (5-fold block local reconstruction), EMD-35183 [https://www.ebi.ac.uk/emdb/EMD-35183] and 8I4T [https://doi.org/10.2210/pdb8i4t/pdb] (composite map of the ASU and the atomic model), EMD-35540 [https://www.ebi.ac.uk/emdb/EMD-35540] and 8ILQ [https://doi.org/10.2210/pdb8ilq/pdb] (locally averaged density of Gn/Gc heterodimer and the atomic model). The subtomogram averaging map of the hexon was deposited as an additional map under the same entry of the ASU map. Source data are provided with this paper.

Human research participants

Policy information about studies involving human research participants and Sex and Gender in Research.

Reporting on sex and gender	N/A
Population characteristics	N/A
Recruitment	N/A
Ethics oversight	N/A

Note that full information on the approval of the study protocol must also be provided in the manuscript.

Field-specific reporting

Please select the one below that is the best fit for your research. If you are not sure, read the appropriate sections before making your selection.

☒ Life sciences ☐ Behavioural & social sciences ☐ Ecological, evolutionary & environmental sciences

For a reference copy of the document with all sections, see nature.com/documents/nr-reporting-summary-flat.pdf

Life sciences study design

All studies must disclose on these points even when the disclosure is negative.

Sample size	No statistical methods were used to predetermine sample size. The sample size of cryo-EM data was chosen based on the sufficient number of particles to achieve high-resolution reconstructions. For the Western blot, three different batches of samples were prepared to replicate the experiment which produced similar results. For pseudovirus infection assays, three batches of samples were prepared to replicate the experiment, and each sample was tested with 6 replicas to enhance the signal-to-noise ratio of the measurements. All three batches of samples produced similar results.
Data exclusions	No data were excluded from analyses.
Replication	All replicas of data produced similar results. The numbers of experiments are described in the Methods section or figure legend where applicable.
Randomization	This is not relevant to our study, because no grouping was needed.
Blinding	Investigators were not blinded to group allocation, as no grouping was needed for this study.

Reporting for specific materials, systems and methods

We require information from authors about some types of materials, experimental systems and methods used in many studies. Here, indicate whether each material, system or method listed is relevant to your study. If you are not sure if a list item applies to your research, read the appropriate section before selecting a response.

Materials & experimental systems

n/a	Involved in the study	
☐	☒	Antibodies
☐	☒	Eukaryotic cell lines
☒	☐	Palaeontology and archaeology
☒	☐	Animals and other organisms
☒	☐	Clinical data
☒	☐	Dual use research of concern

Methods

n/a	Involved in the study	
☒	☐	ChIP-seq
☒	☐	Flow cytometry
☒	☐	MRI-based neuroimaging

Antibodies

Antibodies used	SFTSV HB29 polyclonal antibody (Catalog #PAB27171, Abnova, 1:500)<br>Monoclonal ANTI-FLAG® M2 antibody (Clone M2, Catalog #F3165, Sigma-Aldrich, 1:50,000)<br>Monoclonal anti-Beta Actin antibody (Catalog #66009-1-Ig, Proteintech, 1:2000)<br>HRP-conjugated Goat Anti-Rabbit IgG (H+L) (Catalog #A0208, Beyotime, 1:2000)<br>HRP-conjugated Goat Anti-Mouse IgG (H+L) (Catalog #A0216, Beyotime, 1:2000)
Validation	SFTSV HB29 polyclonal antibody: https://www.abnova.com/protocol_pdf/DS_PAB27171.pdf<br>Monoclonal ANTI-FLAG® M2 antibody: https://www.sigmaaldrich.cn/deepweb/assets/sigmaaldrich/product/documents/415/240/f3165dat-mk.pdf<br>Monoclonal anti-Beta Actin antibody: https://www.sigmaaldrich.cn/deepweb/assets/sigmaaldrich/product/documents/415/240/f3165dat-mk.pdf<br>HRP-conjugated Goat Anti-Rabbit IgG (H+L): https://www.beyotime.com/Manual/A0208_HRP-labeled%20Goat%20Anti-Rabbit%20IgG(H+L).pdf<br>HRP-conjugated Goat Anti-Mouse IgG (H+L): https://www.beyotime.com/product/A0216.htm

Eukaryotic cell lines

Policy information about cell lines and Sex and Gender in Research

Cell line source(s)	African green monkey kidney (Vero), HEK293T/17 and HEK293 cells were used for virus propagation, pseudovirus packaging and infection assays. All cells were donated by the Stem Cell Bank, Chinese Academy of Sciences.
Authentication	The cells were obtained from the Stem Cell Bank, Chinese Academy of Sciences, and routinely maintained in our lab. They were not authenticated experimentally for these studies.
Mycoplasma contamination	All cell lines were tested negative for mycoplasma contamination.
Commonly misidentified lines<br>(See ICLAC register)	No misidentified cell lines were used in this study.
