# Supplementary figures and images for: Cryo-EM structure of severe fever with thrombocytopenia syndrome virus
Source: Nat Commun. 2023 Oct 10;14:6333. doi: 10.1038/s41467-023-41804-7 (PMC10564799; doi:10.1038/s41467-023-41804-7)

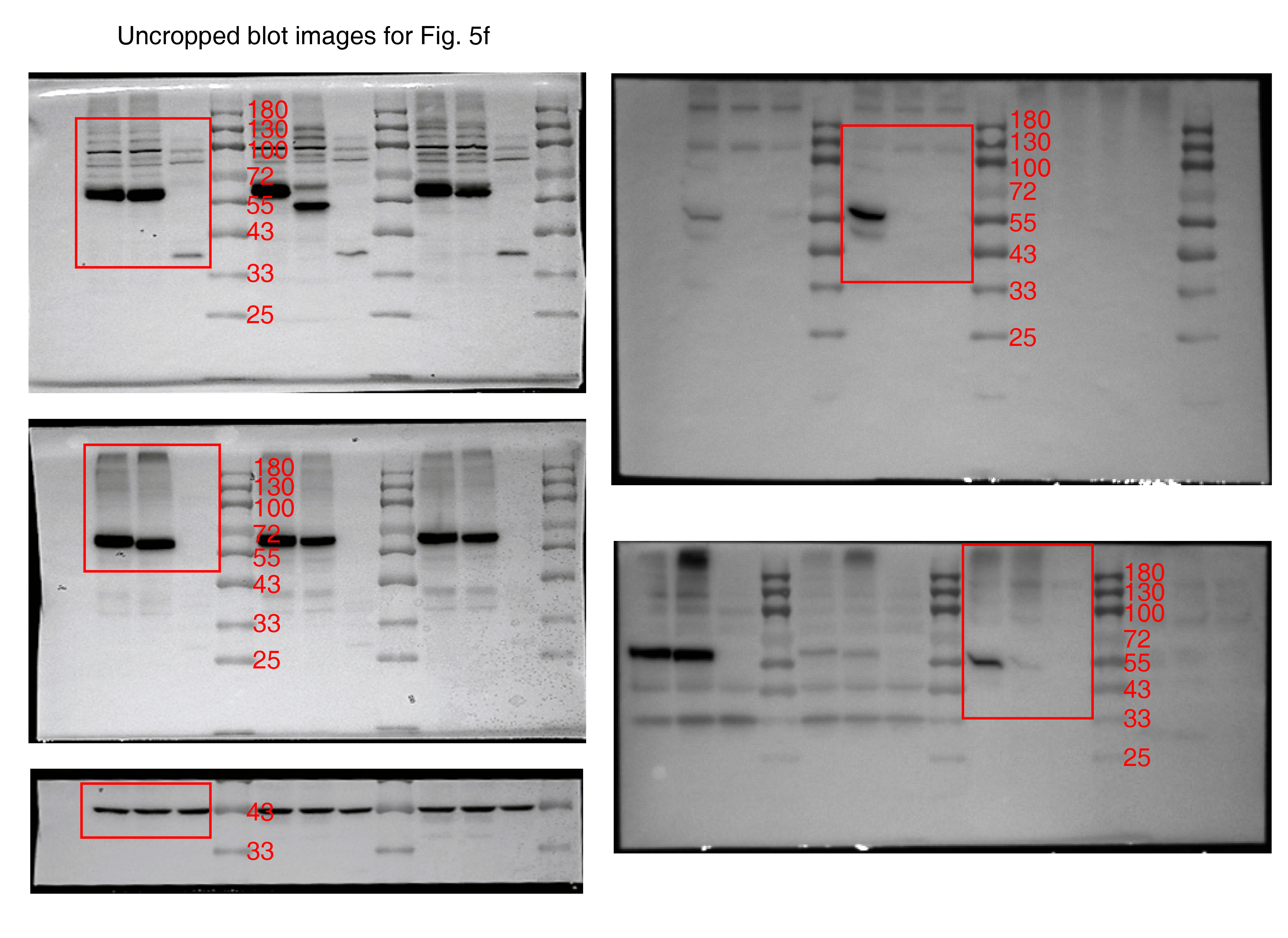

Supplement: Supplementary file 4 — Source Data [file 41467_2023_41804_MOESM4_ESM.zip › Source Data blots.tif]
